# Histology of human myocardial laminar microstructure and consideration of its cyclic deformations with respect to interpretation of *in vivo* cardiac diffusion tensor imaging

**DOI:** 10.1186/1532-429X-17-S1-Q10

**Published:** 2015-02-03

**Authors:** Philip J Kilner, Karen McCarthy, Margarita Murillo, Pedro Ferreira, Andrew D Scott, Laura-Ann McGill, Sonia Nielles-Vallespin, Ranil Silva, Dudley J Pennell, Siew Y Ho, David Firmin

**Affiliations:** 1 CMR Unit, Royal Brompton Hospital, London, UK; 2 National Heart and Lung Institute, Imperial College, London, UK

## Background

Cardiovascular magnetic resonance (CMR) diffusion tensor imaging (CDTI) shows potential to interrogate myocardial microstructure and its dynamics in health and disease. It has been suggested that anisotropies of diffusion measured *in vivo* might be accounted for and need correction by myocardial strains measured macroscopically. However, relations between nano-, micro- and macro-scale deformations are complex in myocardium. Appreciation of the nature and functional role of myocardial laminar microstructures (sheetlets and shear layers) seems sparse and we have been unable to find appropriate visualisation of them in human hearts. We aimed to visualise the microstructures which could logically account for anisotropies of the ~50μm mean distances of diffusion per cycle recorded by CDTI in humans, and to conceptualise the likely effects on equeous diffusion of their cyclic re-orientations and deformations.

## Methods

In three human hearts (unused transplant donor) and two pig hearts, all formalin fixed, we made short and long axis cuts to remove a full thickness rectangular block from the mid lateral wall. Blocks were divided into 5 slices, parallel to the epicardial surface. Each slice was then cut perpendicular to its myocyte orientation, identified by surface grain of its wall tangent faces. After marking faces for reference, the pieces were prepared for sectioning perpendicular to myocytes. The trichrome stained sections were micro-photographed and displayed to show the distribution and orientations of sheetlets and shear layers through the wall (Figure). Based on them, we constructed physical models using straws and pivoted slats to help conceptualise their movements during contraction.

**Figure 1:**
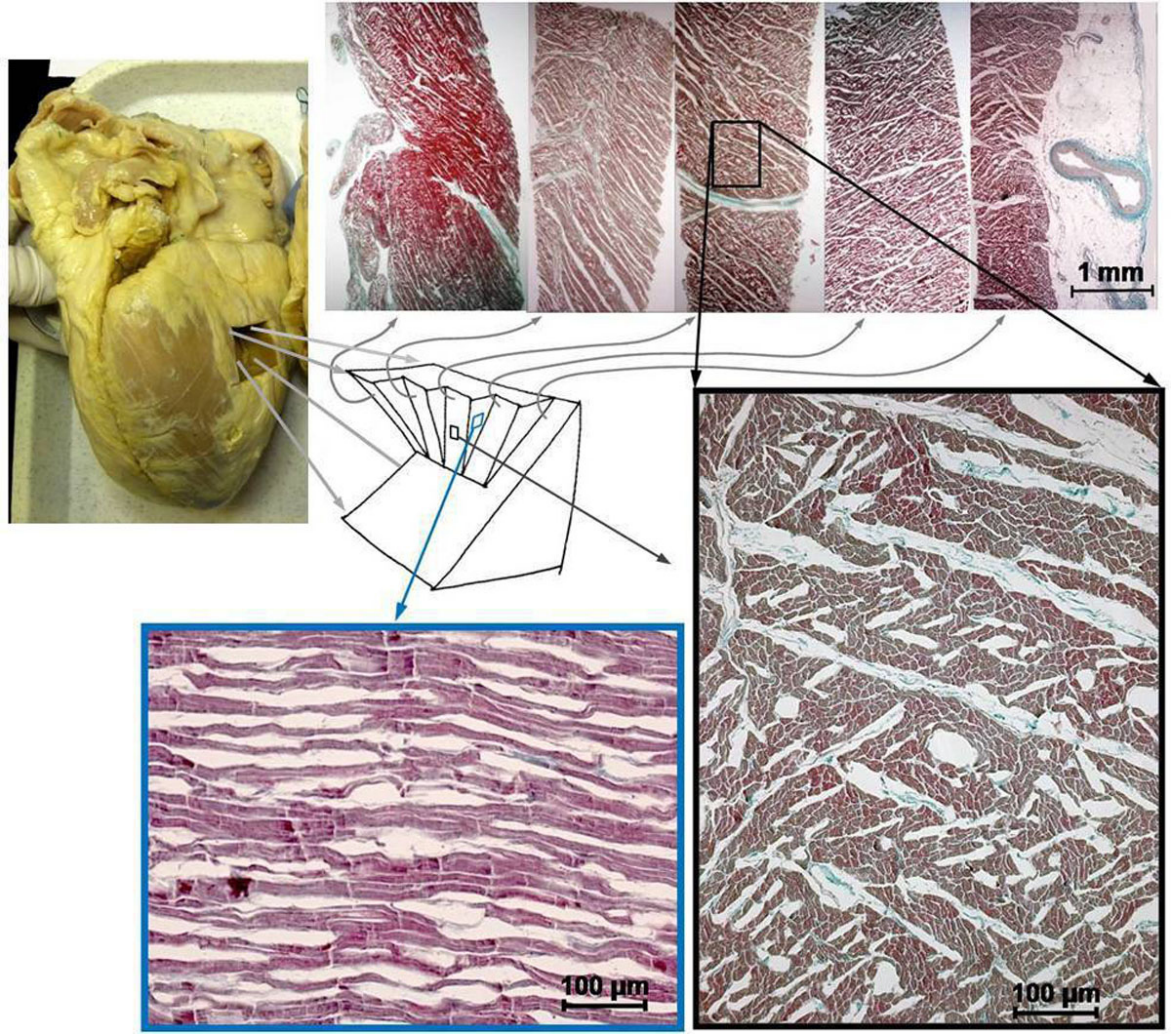
Human myocardial histology. Five tangential slices of a mid lateral block were each sectioned perpendicular to the local myocytes to reveal laminar microstructure (above). Individual myocytes can be distinguished at high magnification of the mid wall cross-myocyte section (below right) and a wall tangent section (below left). Shear layers (cracks) appear to have gaped post mortem in this heart, which enhances their visibility.

**Figure 2:**
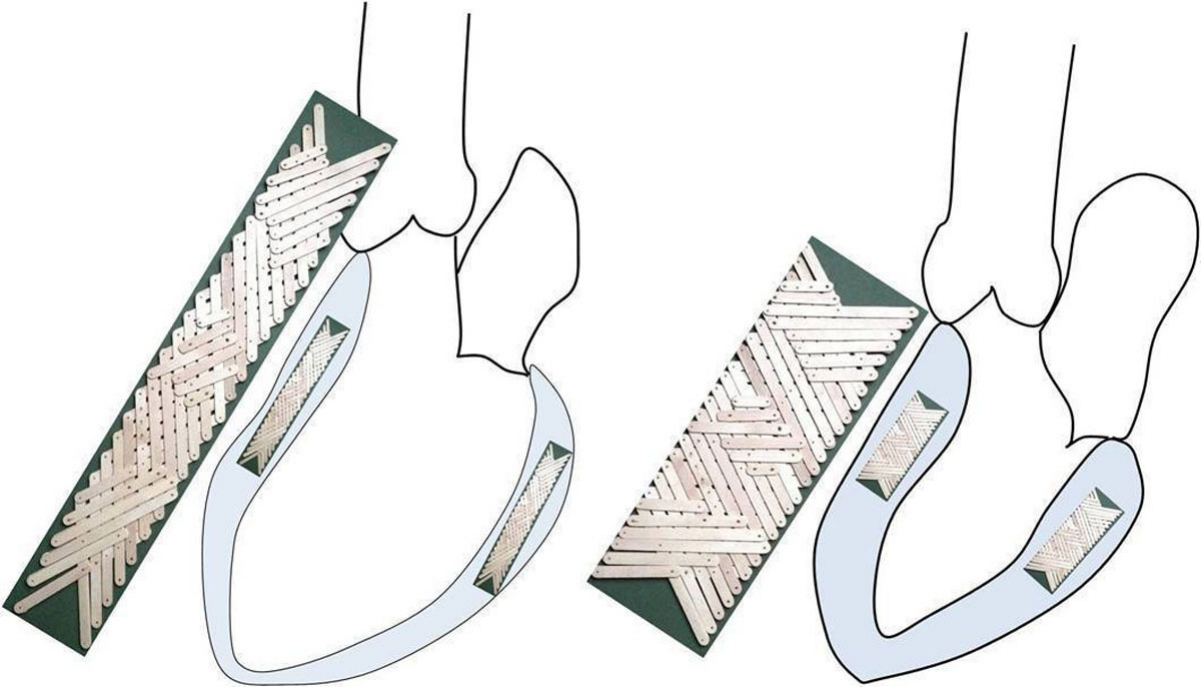
Pivoted lattice model, not to scale, offering an illustration of the reorientations of populations of sheetlets and shear layers of the midwall from diastole (left) to systole (right). Myocytes run approximately circumferentially through the mid wall. Reorientating laminae accommodate systolic cross-myocyte shortening, in spite of their individual thickening, and translate it to enhanced wall thickening. In the midwall, laminar reorientations are driven by the contraction of the more longitudinally orientated myocytes of more epicardial and endocardial layers.

## Results

Nearly all of the depth of compact myocardium of all 5 hearts showed laminar microstructure, with myocytes of ~12-20µm diameter aggregated in sheetlets of ~80μm thickness with shear layer interstices between. The papillary muscles, trabeculations and the immediate subepicardial layer generally lacked laminar structure. Our models helped us conceptualise reorientations of sheetlet and shear layer sub-populations, swivelling from more wall-parallel in diastole to more wall-perpendicular in systole, with associated shearing of extracellular fluid in interstices between.

## Conclusions

The above microstructures, notably the shear layers, their mean intravoxel alignments and their dynamics are likely to underlie the anisotropies of aqueous diffusion recorded *in vivo* by CDTI. It would be illogical to ‘correct' these using strain data acquired macroscopically by CMR, particularly in the cross-myocyte directions. The directions, magnitudes and even signs of strains differ between micro-compartments and between scales. The high cross-myocyte systolic strains of wall thickining and subendocadial circumferential shortening, for example, have been shown to be accounted for more by laminar reorientations, with interstitial shearing, than by the thickening and compression of individual myocytes.

## Funding

National Institute of Health Research Cardiovascular Biomedical Research Unit, Royal Brompton Hospital and Imperial College, London. British Heart Foundation.

